# Viral dissemination and immune activation modulate antiretroviral drug levels in lymph nodes of SIV-infected rhesus macaques

**DOI:** 10.3389/fimmu.2023.1213455

**Published:** 2023-09-18

**Authors:** Sharat Srinivasula, Paula Degrange, Simone Perazzolo, Andrew Bonvillain, Amanda Tobery, Jacob Kaplan, Hyukjin Jang, Refika Turnier, Michael Davies, Mackenzie Cottrell, Rodney J. Y. Ho, Michele Di Mascio

**Affiliations:** ^1^ AIDS Imaging Research Section, Clinical Monitoring Research Program Directorate, Frederick National Laboratory for Cancer Research, Frederick, MD, United States; ^2^ AIDS Imaging Research Section, Charles River Laboratories, Integrated Research Facility, National Institute of Allergy and Infectious Diseases (NIAID), National Institutes of Health (NIH), Frederick, MD, United States; ^3^ Department of Pharmaceutics, University of Washington, Seattle, WA, United States; ^4^ AIDS Imaging Research Section, Division of Clinical Research, National Institute of Allergy and Infectious Diseases (NIAID), National Institutes of Health (NIH), Poolesville, MD, United States; ^5^ Clinical Support Laboratory, Frederick National Laboratory for Cancer Research, Frederick, MD, United States; ^6^ Division of Pharmacotherapy and Experimental Therapeutics, University of North Carolina Eshelman School of Pharmacy, Chapel Hill, NC, United States; ^7^ Department of Bioengineering, University of Washington, Seattle, WA, United States

**Keywords:** SIV infection, lymph nodes, immune activation, drug metabolite, rhesus macaque, tenofovir, pharmacokinetic model, antiretroviral therapy (ART)

## Abstract

**Introduction and methods:**

To understand the relationship between immunovirological factors and antiretroviral (ARV) drug levels in lymph nodes (LN) in HIV therapy, we analyzed drug levels in twenty-one SIV-infected rhesus macaques subcutaneously treated with daily tenofovir (TFV) and emtricitabine (FTC) for three months.

**Results:**

The intracellular active drug-metabolite (IADM) levels (TFV-dp and FTC-tp) in lymph node mononuclear cells (LNMC) were significantly lower than in peripheral blood mononuclear cells (PBMC) (P≤0.005). Between Month 1 and Month 3, IADM levels increased in both LNMC (P≤0.001) and PBMC (P≤0.01), with a steeper increase in LNMC (P≤0.01). The viral dissemination in plasma, LN, and rectal tissue at ART initiation correlated negatively with IADM levels at Month 1. Physiologically-based pharmacokinetic model simulations suggest that, following subcutaneous ARV administration, ART-induced reduction of immune activation improves the formation of active drug-metabolites through modulation of kinase activity and/or through improved parent drug accessibility to LN cellular compartments.

**Conclusion:**

These observations have broad implications for drugs that need to phosphorylate to exert their pharmacological activity, especially in the settings of the pre-/post-exposure prophylaxis and efficacy of antiviral therapies targeting pathogenic viruses such as HIV or SARS-CoV-2 replicating in highly inflammatory anatomic compartments.

## Introduction

The two most commonly used nucleos(t)ide reverse transcriptase inhibitors (NRTIs), tenofovir (TFV) and emtricitabine (FTC), in antiretroviral (ARV) therapy (ART) for HIV-infection in humans are also the ones, but only in combination with one another, approved for daily use as pre-exposure prophylaxis (PrEP) of HIV prevention strategy. In HIV-infection in humans and SIV-infection of rhesus macaques (RMs), lymphoid tissues are a major site of viral production and storage (1). Despite effective ART from the perspective of subdued plasma viremia, the prevailing consensus is that ART is unable to eliminate latent viral reservoirs and also fails to fully suppress viral replication in peripheral lymphoid tissues (2–6). Both inadequate spatial ARV exposure and suboptimal total active-drug concentrations in reservoir tissues were hypothesized as a basis for spawning the organs into viral sanctuaries of relentlessly active low-level virus production during ART. To this end, studies found anatomically heterogeneous ARV drug distribution in reservoir tissues (7–10) and significantly lower concentrations of the pharmacologically active metabolites of TFV (TFV-diphosphate, TFV-dp) and FTC (FTC-triphosphate, FTC-tp) in peripheral lymph nodes than in peripheral blood (11–14), although it is unknown whether those levels are sufficiently high enough to produce an optimal antiviral activity in the lymph node compartment (15, 16) or in other lymphoid organs (e.g. the spleen) (17, 18) in humans.

Understanding factors that regulate penetration of NRTIs across the peripheral blood-lymphatic barrier and into lymph nodes, their availability to cells, phosphorylation within the cell, and their clearance can help develop novel therapeutic formulations such as lipid-based nanoparticles optimized for HIV treatment and prevention (19–21). To study the relationship between immunovirological factors and drug levels in lymph nodes, we analyzed data from a dataset that was originally conceived as part of a larger study designed to evaluate anti-α_4_β_7_ monoclonal antibody therapy in the control of SIV-infection (22).

## Materials and methods

### Animals, study design, and antiretroviral therapy

Twenty-one of twenty-two Indian rhesus macaques (RMs) (*Macaca mulatta*) described in Di Mascio et al. (22) were used in this study following the National Institute of Allergy and Infectious Diseases (NIAID) Animal Care and Use Committee approved protocol. This study excluded one animal from the original twenty-two because we were unable to collect lymph node (LN) tissue during the animal’s biopsy procedures. Briefly, twenty-one juvenile to adult RMs (10 female, 11 male; age at recruitment: median 5.4 years (range 2.6-17.3); weight at recruitment: median 7.8kg (range 3.8-12.8)), negative for Mamu A001, B008, and B017 alleles (the alleles associated with spontaneous control of SIV) were inoculated intravenously with 200 TCID_50_ SIVmac239-*nef-stop*. After five weeks of acute SIV-infection, all twenty-one animals were antiretroviral (ARV) treated for 13 weeks (91 days) (**Supplementary Figure 1**). Administered once daily, the antiretroviral therapy (ART) regimen consisted of three ARV drugs: two nucleos(t)ide reverse transcriptase inhibitors (provided by Gilead) tenofovir (TFV, 20mg/kg subcutaneous), and emtricitabine (FTC, 30mg/kg subcutaneous), and an integrase strand transfer inhibitor (provided by Merck) L-870812 (20mg/kg (max 100mg), oral, mixed in food). Stock solutions of TFV and FTC were prepared once every two weeks in sterile water, stored at 4°C, and injected subcutaneously in the scapular area of the back. For the drug mixed in food, macaques were observed to ensure the drug was taken.

Between Day 28 and Day 91 of ART, starting on Day 28 and administered once every three weeks, animals received three intravenous infusions of 50mg/kg of either a primatized anti-α_4_β_7_ monoclonal antibody (mAb) (n=11) or an isotype-matched control mAb (n=10).

For logistic feasibility and to maintain rigorous timings, animals were split into four study groups (five or six animals per group), with each group starting the study two weeks apart. All animal experiments and processing were carried out under a rigid schedule and by the same lab personnel. All procedures were performed at the National Institutes of Health (NIH) Animal Center. Animals’ health was monitored daily and are cared for according to the *Guide for the Care and Use of Laboratory Animals*, 8^th^ Ed., Animal Welfare Act regulations, and policies of NIH, an AAALAC-accredited facility. Macaques were individually housed in 18 cu ft stainless steel cages on a 12-hour light/dark cycle in a temperature-controlled indoor facility with access to behavioral enrichment toys and were fed Purina Old World Primate Diet with rotating enrichment food. Animals were anesthetized with standard doses of Ketamine or Telazol for procedures considered to cause pain or distress to humans.

### Blood, lymph node, and rectal sample collection and processing

To monitor peripheral blood (PB) cell counts and plasma viremia, PB was collected at baseline (pre-viral inoculation) and weekly for the first nine weeks after viral inoculation and then every three weeks thereafter.

For ARV measurement, plasma, PB mononuclear cells (PBMC), and LN tissue samples were collected at two timepoints - on Day 28 and Day 91 of daily ART. The animals were bled and biopsied just before the collection day’s ARV administration, i.e., 24 hours post-dose. The LN biopsy procedure generally lasted 30-40 min, during which time the blood for plasma and PBMC was collected from the femoral vein in Cell Preparation Tube (BD Vacutainer CPT). Post centrifugation at room temperature for 30 min at 1600xg, plasma was immediately removed and stored at -80°C, and PBMC were isolated from the buffy coat. The buffy layer was transferred to a 15mL centrifuge tube and washed with 10mL of phosphate-buffered saline (PBS) at 400xg for 5 min. After lysing the red blood cells for 1 min using ACK lysing buffer, cells were rewashed, resuspended in 1mL PBS for counting with a hemocytometer, and pelleted into aliquots of 1-2 million viable cells.

On Day 28 (M1), the left axillary LN was harvested, and on Day 91 (M3), the right inguinal LN was collected. The surgically excised LN was divided into pieces for use. The first preference was given to storing a piece at -80°C for the measurement of tissue levels of SIV-RNA and SIV-DNA. Next, a portion of tissue was used to isolate LN mononuclear cells (LNMC) by mechanically disrupting by pressing it with the rubber end of a syringe plunger through a 100µm pore size nylon mesh cell strainer (Corning). LNMC were transferred to a 15mL centrifuge tube and washed with 10mL of PBS at 400xg for 5 min. After lysing the red blood cells for 1 min using ACK lysing buffer, cells were rewashed, resuspended in 1mL PBS for counting with a hemocytometer, and aliquoted into pellets of 1 million viable cells. To prevent the degradation of intracellular active-drug metabolites (IADM), PBMC and LNMC cell pellets were snap-frozen in liquid nitrogen and stored at -80°C until analysis. Finally, when enough LN tissue was obtained, a piece was secured in aluminum foil, snap-frozen in liquid nitrogen, and stored at -80°C until ARV drug and endogenous nucleotide analysis of tissue homogenate. Because of the order of priority and the amount of LN tissue collected within a reasonable biopsy time, we have n=21 for lymph node SIV-RNA and -DNA levels, n=21 for IADM levels in LNMC, but only n=12 (at M1) and n=13 (at M3; the same 12 animals of M1 plus one additional animal) for the data extracted from LN tissue homogenates (Subgroup A).

In addition to LN tissue collections at M1 and M3 of daily ART, a right axillary LN biopsy was performed on the day before ART initiation (M0). Along with the LN biopsy, five rectal pinches were also collected using 3mm round jaw biopsy forceps at all three timepoints and stored at -80°C until the measurement of tissue SIV-RNA and SIV-DNA levels (**Supplementary Figure 1**).

Due to the original design constraints (22), to collect LN tissue at every biopsy timepoint, either axillary or inguinal LN clusters were strategically targeted. While drug levels from different LN clusters were evaluated, the parent TFV and FTC concentrations and their IADM levels in LNs of RMs have been shown to be not significantly different among different LN clusters (16, 23).

### Quantification of drug levels in plasma, cells, and tissue

In plasma, TFV and FTC concentrations (ng/mL) were measured. In PBMC, intracellular TFV-diphosphate (TFV-dp) and FTC-triphosphate (FTC-tp), the pharmacologically active moieties of TFV and FTC, respectively, were measured. LN drug levels were investigated using two methods: by isolating LNMC and by tissue homogenization. In LNMC, TFV-dp and FTC-tp levels (femtomoles (fmol) per million cells) were measured. In LN tissue homogenate, parent TFV and FTC concentrations (ng/g), TFV-dp and FTC-tp concentrations (picomoles/g), and concentrations of three endogenous nucleotides - deoxyadenosine triphosphate (dATP), deoxycytidine triphosphate (dCTP), and deoxyguanosine triphosphate (dGTP), quantified as picomoles/g of tissue - were measured.

Following protein precipitation with isotopically labeled internal standards, the plasma, PBMC/LNMC lysates, and tissue homogenate samples were extracted by adapting liquid chromatography with tandem mass spectrometry (LC-MS/MS) methods previously published (15). Calibration standards and quality control samples were prepared in matched blank matrices for the plasma and tissue assays or in 70:30 methanol:water for the cell assay. Precision and accuracy were within 20% for the following calibrated ranges: plasma TFV and FTC 2-2000ng/mL, cell lysate TFV-dp and FTC-tp 0.02-20ng/mL, and tissue homogenate TFV and FTC 0.4-500ng/mL, TFV-dp and FTC-tp 0.1-100ng/mL, and dATP, dCTP, and dGTP 0.2-1000ng/mL.

With cells lysed in 0.3mL, and the dynamic range of cell lysate assay from 0.02-20ng/mL, for a sample of one million cells, the lower limits of quantification for TFV-dp and FTC-tp were 13.4 and 12.3 fmol/million cells, respectively. TFV and FTC concentrations in LN tissue were converted from ng/g to ng/mL of tissue using LN tissue density of 1.03g/mL (24). The metabolite-to-endogenous-nucleotide ratios (MERs) were calculated by dividing the active metabolites with their corresponding endogenous nucleotide concentrations, both obtained from LN tissue homogenate (**Supplementary Table 4**). When ratios of covariates are provided in the tables, the ratios are first calculated for each animal and then the descriptive statistics of these ratios are shown.

### Lymphocyte immunophenotyping, plasma, and tissue-associated viral load

EDTA-treated fresh blood samples were stained for CD3, CD4, CD8, CD20, and Ki67, and analyzed by flow cytometry as previously described (25). Plasma SIV-RNA was measured using a gag-targeted quantitative real-time/digital RT-PCR assay with a lower limit of quantification of 3 copies/mL as previously described (26, 27). LN and rectal tissue SIV-RNA and SIV-DNA were quantified using gag-targeted, nested quantitative hybrid real-time/digital RT-PCR and PCR assays and expressed as SIV-RNA or DNA copies per 10^6^ diploid genome cell equivalents as previously described (26, 27).

### Estimation of viral decay rates

A two-phase exponential decay model previously described (28) was used to estimate the decay rates of the rapid first phase and the slower second phase of plasma viremia. For each macaque, plasma viral load data during the 13 weeks of ART (9 timepoints) was best fitted to the model using a maximum-likelihood procedure that allows for censored data (29). The initial viral load of the model was fixed at the measured value at ART initiation, and the viral load that measured below 3 copies/mL was considered censored data in the range of 1 to 3 copies/mL. Model fitting was carried out by coding in LabVIEW 2013 (National Instruments, TX).

The decay rates of SIV-RNA and SIV-DNA in LN and rectal tissues were estimated by linear regression of the natural logarithm of the size of SIV-RNA and DNA pools in tissues using GraphPad Prism 8.2.

### Statistical analysis

Paired samples were compared with the non-parametric Wilcoxon signed-rank test, unpaired samples were compared with the non-parametric Wilcoxon rank-sum test, and associations were assessed with Spearman rank correlation and multivariate regression using Winstat software (Cambridge, MA). A P-value <0.05 was considered statistically significant.

### Physiologically-based pharmacokinetic model of tenofovir and its pharmacologically active moiety

**Figure 1:**
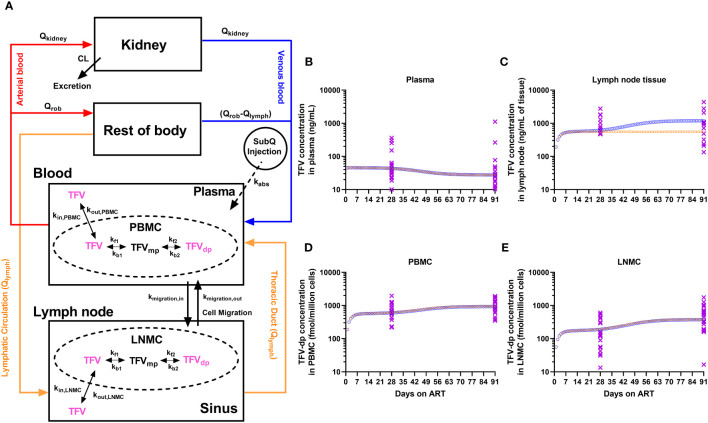
**(A)** Physiologically-based pharmacokinetic (PBPK) modeling of TFV and its pharmacologically active moiety, TFV-dp. The model has a minimized vascular system for the eliminating organ (kidney), and the combined distributing organ (Rest-of-body, ROB). Lymph formation occurs from ROB (e.g., muscles) which drains via lymphatic capillaries to the lymphatic system. The lymphatic system was collapsed into a single representative compartment and divided into sinus and LNMC. NRTIs enter the lymph node by lymphatic circulation and through mononuclear cell migration. Inside the mononuclear cells, the NRTIs undergo kinase-mediated phosphorylation into active moieties. The trough concentrations of TFV and TFV-dp from the PBPK model simulation of a typical macaque (orange open circles for increased kinase-mediated phosphorylation rates; blue open circles for increased LNMC uptake of TFV) and the experimental observations from the 21 macaques (purple crosses) during the 3 months of antiretroviral therapy in the **(B)** plasma, **(C)** lymph node tissue, **(D)** peripheral blood mononuclear cells (PBMC), and **(E)** lymph node mononuclear cells (LNMC).

The physiologically-based pharmacokinetic (PBPK) model schematic of TFV and the phosphorylation cascade to its pharmacologically active intracellular moiety is illustrated in **Figure 1A**. This model was simplified from a novel and extended PBPK model recently proposed by *Perazzolo et al.* (30) and properly validated for TFV in nonhuman primates, which included a more complex representation of the lymphatic system and its communication with other anatomic compartments. The reduced PBPK model here consisted of seven compartments: subcutaneous space, blood (plasma and PBMC), lymph nodes (sinus and LNMC), kidney, and rest-of-body. Briefly, the absorption of TFV into the blood from the subcutaneous injection site was considered fast and complete in NHP (Viread Label, Gilead Sciences, Inc.). Since <1% of the subcutaneous dose goes directly to the lymphatics (30), the direct lymphatic uptake was ignored.

In the blood, PBMC were modeled as an independent compartment in exchange for TFV in the plasma. The plasma-to-PBMC exchange kinetics represents a linear cellular transport of TFV in and out of the cell (*k_in,PBMC_*, *k_out,PBMC_*). Inside the PBMC, parent TFV undergoes a kinase-mediated phosphorylation cascade to an active moiety. Phosphorylation kinetics were modeled on biochemical parameters from *in-vitro* PBMC experiments, as per *Dixit et al.* (31), and scaled in the PBPK context, as per *Perazzolo et al.* (30).

The parent ARV entering the lymph nodes was modeled in two ways: (i) physiological lymph transport (*Q_lymph_*) from the organs (the lymphatic circulation), (ii) trafficking of mononuclear cells via high endothelial venules (HEVs). It is expected that since NRTIs distribute in the interstitial fluid of all organs, they have access to lymphatic recirculation to return to the blood via the thoracic duct. Trafficking of mononuclear cells is broadly observed and greatly occurs via the HEV barrier (32). In the lymph node, LNMC were modeled as an independent compartment in exchange for TFV in the lymph sinus. And similar to plasma-to-PBMC exchange in the blood, TFV in LN can be transported in and out of LNMC (*k_in,LNMC_*, *k_out,LNMC_*) from the sinusoidal space of the node (sinus). Also in LNMC, TFV undergoes phosphorylation at the same rates as in PBMC. In addition to parent ARV, cell migration (*k_migration,in_*, *k_migration,out_*) via HEVs also carry the phosphorylated content between LN and blood.

A set of ordinary differential equations describe the PBPK system (**Supplementary**). The bolus NRTI dose is the input into the subcutaneous space (equation 1). The evolution of concentrations in all the compartments was given in equations 2-11 with initial conditions set to zero. The compartment volumes, flow rates, and pharmacokinetic parameters and their values for a typical nonhuman primate were imported from *Perazzolo et al.* (30) and reported in **Supplementary Table 5**. The model construction and simulations were carried out in MATLAB 2020a (The MathWorks, Inc. MA).

## Results

Twenty-one RMs, acutely infected for five weeks with SIVmac239-*nef-stop*, were treated with daily antiretroviral (ARV) therapy (ART) for thirteen weeks. During ART, ARV drug levels were measured at two timepoints – predose on Day 28 (referred to as Month 1 (M1) timepoint) and predose on Day 91 (the last day of ART; Month 3 (M3) timepoint). The concentrations of parent TFV and FTC in the plasma, and the levels of intracellular active drug-metabolites (IADM) of TFV (TFV-diphosphate, TFV-dp) and FTC (FTC-triphosphate, FTC-tp) in PBMC and LNMC were measured in all animals (n=21). Because of the sampling constraints and limitations discussed in the Methods, the parent ARV concentrations in LN tissue homogenates were measured from only a subset of animals (n=12 at M1 and n=13 at M3; subgroup A). Additionally, LN tissue from subgroup A animals was also used to measure the concentrations of endogenous nucleotides - deoxyadenosine triphosphate (dATP) and deoxycytidine triphosphate (dCTP) (analogs of TFV-dp and FTC-tp, respectively), and deoxyguanosine triphosphate (dGTP) as well as the metabolite-to-endogenous nucleotide ratios (MERs). The study design and sample sizes for each analyte and for each matrix are detailed in **Supplementary Figure 1** and **Supplementary Table 1**.

### *Antagonism of α_4_β_7_ integrin* did not affect ARV drug levels

We previously reported (22) that compared to the control arm, the treatment with anti-α_4_β_7_ monoclonal antibody (mAb) infusions in these animals was not associated with prolonged post-treatment suppression of viremia. In this study, we also observed no statistically significant effect of three anti-α_4_β_7_ mAb infusions administered between M1 and M3 on the drug levels measured at M3, on the IADM ratio between peripheral blood (PB) and lymphoid compartments at M3, or on the drug level changes between M1 and M3 (**Supplementary Figure 2**). As a result, we gathered animals from both arms in this study.

### Parent TFV and FTC concentrations in the plasma decreased between month one and month three of daily ART

**Figure 2:**
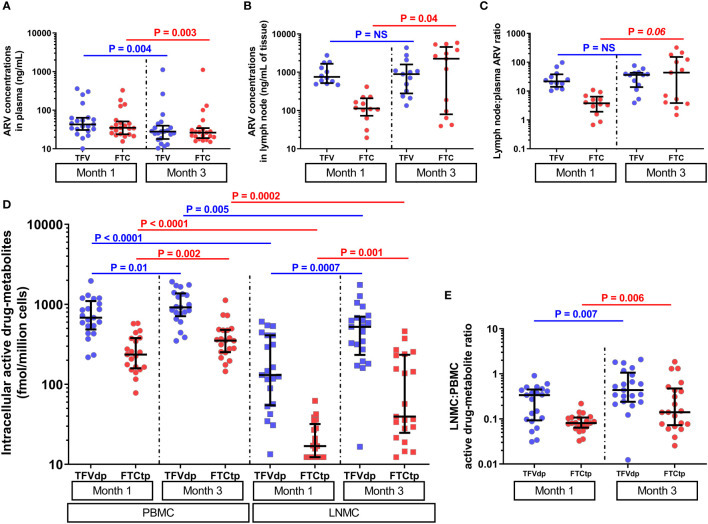
The parent antiretroviral (ARV) concentrations in **(A)** plasma (n=21) and **(B)** lymph node tissue homogenate (subgroup A, n=12 at M1 and n=13 at M3), and **(C)** lymph node tissue to plasma ARV ratios (subgroup A, n=12 at M1 and n=13 at M3) at months 1 and 3 of antiretroviral therapy (ART). The lymph node tissue ARV concentrations were several-fold higher than plasma ARV concentrations (P <0.005, n=12). **(D)** The levels of intracellular active drug metabolites (IADM) in peripheral blood mononuclear cells (PBMC, n=21) and lymph node mononuclear cells (LNMC, n=21), and **(E)** LNMC to PBMC IADM ratios at months 1 and 3 of ART (n=21). The IADM levels in LNMC were statistically significantly lower than in PBMC (P ≤0.005, n=21). Data were summarized with a scatter dot plot with median and interquartile range.

All 42 plasma samples (21 animals x 2 timepoints) analyzed for parent ARVs (expressed as ng/mL) measured above the lower limit of quantification (LLOQ). A high degree of direct correlation between TFV and FTC trough concentrations was observed at both M1 and M3 (ρ >0.67, P ≤0.0005, n=21). Both TFV and FTC trough concentrations decreased at M3 vs. M1 (by a median of 1.6-fold and 1.4-fold, respectively, P <0.005, n=21, **Figure 2A**), with changes during time not statistically significantly correlated with the plasma viral load at ART initiation (P =non-significant, NS).

### TFV-dp and FTC-tp levels significantly increased in both PBMC and LNMC between month one and month three of daily ART

All 42 PBMC samples analyzed for IADM (expressed as fmol/million cells) measured above LLOQ. For 42 LNMC samples, one animal for TFV-dp and nine animals for FTC-tp at M1, none for TFV-dp and one animal for FTC-tp at M3 measured below LLOQ. Samples that measured below LLOQ were imputed at LLOQ.

At both M1 and M3, the parent ARV concentrations in plasma were positively correlated with their respective IADM levels in PBMC (ρ =0.42-0.72, P ≤0.03, n=21) but were uncoupled with LNMC IADM levels (P =NS). Similar to the strong positive correlations observed between the concentrations of TFV and FTC in plasma intracellularly, strong positive correlations were found between TFV-dp and FTC-tp levels in both PBMC and LNMC at both M1 and M3 (ρ =0.54-0.79, P ≤0.005, n=21). A dditionally, PBMC IADM levels were significantly positively correlated with their respective LNMC IADM levels at M1 (ρ ≥0.45, P ≤0.02, n=21) but not at M3 (P =NS).

FTC-tp levels were significantly lower (P <0.0001, n=21) than TFV-dp in both PBMC and LNMC at both M1 and M3 (**Supplementary Table 2**). The IADM levels in LNMC were significantly lower than PBMC at both M1 (median 3-fold (TFV-dp) and 12.3-fold (FTC-tp) lower, P <0.0001, n=21) and M3 (median 2.3-fold (TFV-dp) and 7.1-fold (FTC-tp) lower, P ≤0.005, n=21) (**Figure 2D**). Between M1 and M3, TFVdp and FTCtp levels increased in both LNMC (by median 2.1-fold and 2.9-fold, respectively, P *≤*0.001, n=21) and PBMC (by median 1.4-fold and 1.5-fold, respectively, P ≤0.01, n=21) (**Figure 2D**). Though the median LNMC IADM levels were still significantly lower than PBMC levels at M3, the accumulation of active drug-metabolites in LNMC was much steeper than the increase in PBMC, as evidenced by a statistically significant increase in the LNMC to PBMC molar ratio for both TFVdp (median 2.2-fold, P =0.007, n=21) and FTCtp (median 2.7-fold, P =0.006, n=21) (**Figure 2E**).

### Parent TFV and FTC concentrations in LN tissue of subgroup A were several-fold higher than in plasma with an inconclusive trend during time on ART

The median (interquartile range [IQR]) plasma TFV and FTC trough concentrations (n=21) at M1 were 42.9ng/mL (IQR: 30.6-63.6) and 34.9ng/mL (IQR: 23.9-51.3), respectively, and in LN tissue (n=12) were 754.9ng/mL (IQR: 520.1-1664.8) and 113.3ng/mL (IQR: 73.0-208.6), respectively (**Figures 2A, B**). Using matched plasma and LN tissue homogenate samples at M1, we observed that the LN ARV concentrations were several-fold higher than plasma ARV concentrations (P <0.005, n=12) (**Figure 2C** and **Supplementary Table 3**). Between M1 and M3, a slight but not statistically significant increase in TFV (1.1- fold) and a statistically significant increase in FTC concentration (median 20- fold, P =0.04) was observed in LN tissue

### A mechanistic hypothesis to explain parent drug and metabolite levels via PBPK modeling

**Figure 3:**
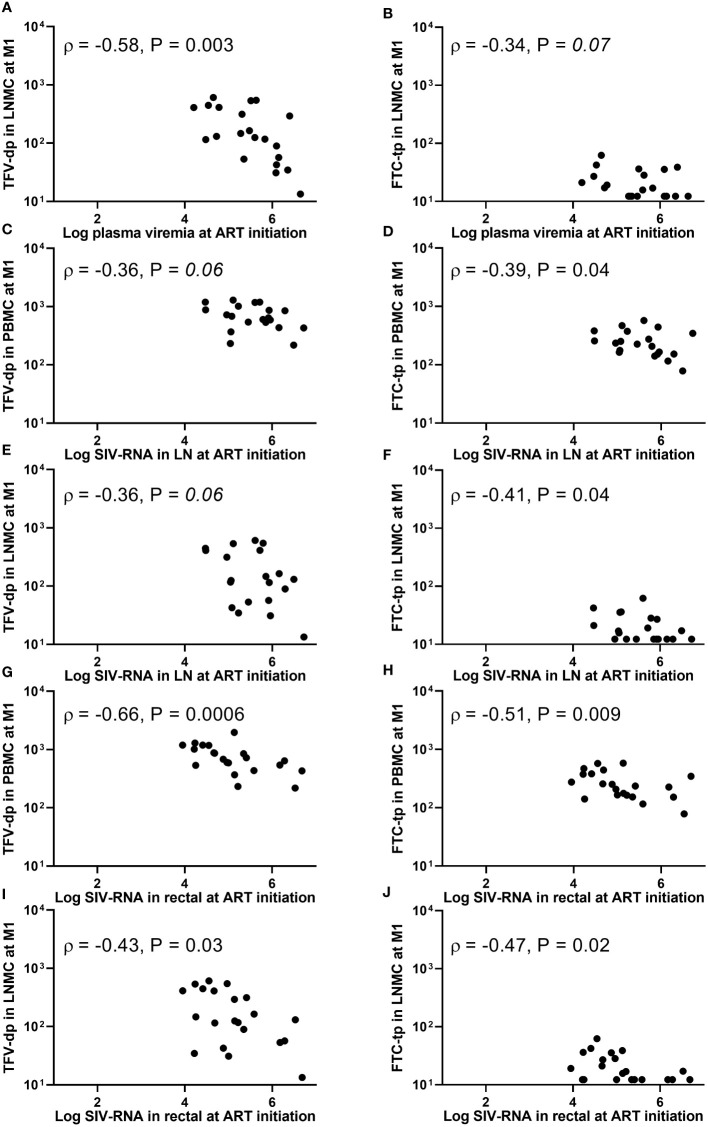
Associations between **(A, B)** plasma viremia (SIV-RNA copies/mL), **(C-F)** SIV-RNA levels (copies/10^6^ cell equivalent) in lymph node tissue, or **(G-J)** SIV-RNA levels (copies/10^6^ cell equivalent) in rectal tissue at the initiation of antiretroviral therapy (ART) and intracellular active drug metabolite levels (fmol/million cells) in peripheral blood mononuclear cells (PBMC) or lymph node mononuclear cells (LNMC) at one month of ART. Viral dissemination at ART initiation was generally inversely associated with intracellular active drug metabolite levels at one month into therapy.

We next investigated if the above dynamics were of pharmacokinetic origin using a physiologically-based pharmacokinetic (PBPK) model (**Figure 1A**) consisting of a set of 11 equations described in the Methods and **Supplementary**. With parameter values in **Supplementary Table 5**, we simulated the model in a typical macaque receiving daily subcutaneous ART to reproduce the trough concentrations of TFV in plasma, TFV-dp in LNMC and PBMC (all 3 outputs experimentally observed from the full dataset of n=21), and TFV in LN tissue (observed only in subgroup A). In subgroup A, we observed trends similar to those observed in the full dataset including a steeper increase of TFV-dp in LNMC compared to PBMC (**Supplementary Figure 3**), however, because of the lower sample size, the slopes of TFV-dp in LNMC and PBMC were not statistically significantly different (**Supplementary Figure 3E**). Additionally, subset A consists of animal subjects with plasma viral load at ART initiation lower than the remaining nine animals (Log_10_ mean ± standard deviation 5.11 ± 0.54 vs. 5.98 ± 0.61 copies/mL; P = 0.004, n=12 vs. n=9), as well as a smaller increase of TFV-dp in LNMC between M1 and M3 compared to the remaining nine animals (median 1.7-fold vs. 6.6-fold, P = 0.016, n=12 vs. n=9). The above trend was in part expected from the inverse correlation between baseline levels of viral dissemination and TFV-dp levels at M1 (**Figure 3**). To this effect, we also observed a negative correlation between TFV-dp levels in LNMCs at M1 and the slope of TFV-dp change in LNMC between M1 and M3 (ρ = -0.60, P = 0.002, n=21). Though the parent ARV accumulation trend in LN tissue is inconclusive and obtained from a smaller sample size, these additional measurements provided information on the absolute levels of parent drug concentrations in LNs and their relation to plasma ARV concentrations (**Figure 2C**), useful to tune the parameters of the model.

The plasma steady-state concentration was achieved within 2 days. The PBMC and LNMC steady-state concentration of TFV-dp, and LN tissue steady-state concentration of TFV was achieved by Day 10 (**Figures 1B-E**). Based on the model configuration, we narrowed down the ART effects on SIV-infection into two most likely scenarios: (i) Increased plasma clearance (*CL*), increased phosphorylation rates (denoted by *k_f1_* and *k_f2_* rate constants), and increased cell migration rates (denoted by *k_migration,in_* and *k_migration,out_* rate constants). (ii) Increased plasma clearance, increased LNMC uptake of ARV in the lymph nodes (denoted by *k_in_LNMC_*), and increased migration rates.

Both scenarios had an increase in plasma *CL* of ~10%, which predicted well the plasma observations. The *CL* change did not affect IADM levels in both PBMC and LNMC. In fact, PBMC and LNMC concentrations were more sensitive to the uptake and intracellular conversion kinetics than plasma *CL* change. In scenario (i), the increase in phosphorylation rates alone led to IADM accumulation in both PBMC and LNMC. Together with changes in cell trafficking rates, scenario (i) could explain our IADM levels and their differential change between M1 and M3 in the two compartments. Here, our model predicted that TFV in LN tissue would reach an early plateau (by Day 10) and remain unchanged during time. In scenario (ii), we assumed that the phosphorylation rates are constant and that there is an improvement in the availability of parent NRTI in the LN for more intracellular conversion. This led to higher LNMC IADM levels, which, along with increased/improved migration rates, could explain the different degrees of TFV-dp increase in PBMC and LNMC. Here, our model predicted an increase of TFV in LN tissue over time with a slope similar to the slope of the LNMC TFV-dp levels (**Figure 1**).

Both scenarios robustly matched the differential TFV-dp dynamics in PBMC and LNMC obtained from the full dataset. Further research is needed to ascertain the dynamics of TFV levels in LN tissue homogenates in a larger group of animals with a range of baseline viral loads wider than Subgroup A. However, exploring the dynamics of early plateau or a putative increase of TFV in LNs between M1 and M3 of ART, our model suggests that either improved LNMC uptake of parent TFV in the lymph node, or increased kinase-mediated phosphorylation rates specific for TFV or a combination of both factors form the mechanistic hypothesis to explain the full dataset in our study.

### Higher viral dissemination was associated with lower intracellular active drug-metabolite levels

The plasma viral load (PVL) at ART initiation (week 5 post-viral inoculation) ranged wide from 1.6 x 10^4^ to 4.4 x 10^6^ SIV-RNA copies/mL. The plasma viremia at ART initiation (but not PB CD4+ T-cell count) was inversely associated with LNMC (but not PBMC) IADM levels at M1 for both TFV-dp (ρ =-0.58, P =0.003, n=21) and FTC-tp (ρ =-0.34, P =0.07, n=21) (**Figures 3A, B**). In addition, SIV-RNA levels in LN and rectal tissue at ART initiation (range 4.5 to 6.7, and 4.0 to 6.7 log_10_ SIV-RNA copies/10^6^ cell equivalent, respectively) were inversely associated with both LNMC and PBMC IADM levels at M1 for both TFV-dp (LN: ρ =-0.36, P =0.06; Rectal: ρ <-0.43, P <0.05 n=21) and FTC-tp (LN: ρ <-0.39, P <0.05; Rectal: ρ <-0.47, P <0.05 n=21) (**Figures 3C-J**).

The PVL at M1 (reduced to <100 copies/mL in 19 of 21 animals, with 6 animals below LLOQ of 3 copies/mL) correlated negatively with both PBMC and LNMC IADM levels at M1 for both TFV-dp (ρ <-0.41, P <0.05 n=21) and FTC-tp (ρ <-0.38, P <0.05 n=21). Similarly, a negative trend was observed for LN SIV-RNA levels at M1 and both LNMC and PBMC TFV-dp levels at M1 (ρ <-0.37, P ≤0.05, n=21). No significant associations were observed with rectal SIV-RNA levels at M1 (P =NS). However, a negative correlation was noted between LMNC IADM levels at one month into therapy and rectal SIV-DNA levels at both ART initiation (ρ <-0.59, P ≤0.002, n=21) and at M1 (ρ <-0.37, P <0.05, n=21).

Moreover, the area under the curve (AUC) of log_10_ PVL during the first month of ART (AUC_0-1month_) strongly correlated negatively with LNMC IADM levels at M1 for both TFV-dp and FTC-tp (ρ =-0.50, P =0.01 and ρ =-0.48, P =0.01, respectively, n=21), but weakly with PBMC (ρ =-0.44, P =0.02 and ρ = -0.34, P =0.07, respectively, n=21). Because by the end of the three months of ART all animals were PVL suppressed (<10 copies/mL in 17 animals, <30 copies/mL in 3 animals, and <500 copies/mL in one animal), associations of PVL with IADM levels at M3 were not assessed. No significant associations were observed between LN or rectal SIV-RNA and LNMC or PBMC IADM levels at M3 (P =NS). As expected, the SIV-DNA in LN at M3 (when the PVL is suppressed in all animals) was correlated positively with PVL at ART initiation (ρ =0.6, P <0.01, n=21), and negatively with the LNMC TFV-dp level at M1 (ρ =-0.55, P <0.01, n=21).

### LNMC active drug-metabolite levels at month one of ART were not associated with viral decay rates

With ART, plasma viremia declined in two phases. The rapid first phase decayed at a median rate of 0.65 day^-1^ (range 0.31 to 1.25) and transitioned to the slower second phase at a median of 13.5 days (range: 7.2-33.0) after ART initiation. As expected, the PVL at ART initiation was not associated with the first phase decay rate (ρ =0.1, P =0.33, n=21). Despite the PVL at ART initiation and AUC_0-1month_ being predictive of LNMC IADM levels after one month of ART, higher LNMC IADM levels at M1 were not associated with faster decay of first phase plasma viremia (P = NS). Since NRTIs inhibit viral replication by competitive binding of IADM with the natural endogenous deoxynucleoside triphosphate for incorporation by viral reverse transcriptase into the growing viral DNA chain, we decided to explore the associations after normalizing the active drug- metabolite concentrations on their respective competing natural dNTPs (metabolite-to-endogenous nucleotide ratio, MER). However, the endogenous nucleotide concentrations in LNs were measured only in subgroup A animals (**Supplementary Table 4**).

12 samples for dATP (6 at M1 and 6 at M3) and 4 samples for dCTP (2 at M1 and 2 at M3) measured below LLOQ and were imputed at LLOQ. All samples for dGTP measured above LLOQ. At both timepoints, the three dNTP concentrations were highly correlated among them (P <0.001, n=12). Also, at both timepoints, dCTP concentrations were significantly higher than dATP, and dGTP was significantly higher than both dATP and dCTP concentrations (P ≤0.002, n=12) (**Supplementary Figure 4**). Furthermore, at both timepoints, the active drug- metabolite concentrations in LN tissue generally correlated positively with endogenous nucleotide concentrations measured at the same time (**Supplementary. Figure 5**). However, there were no statistically significant changes in all three dNTP concentrations between M1 and M3 (P >0.53, n=12).

Still, MERs at M1 were not significantly associated with faster first phase plasma viral decay (P =NS). Of note, higher LNMC IADM levels at M1 were associated with a lower size of the second phase plasma viral reservoir (range 3 x 10^0^ to 3.9 x 10^4^ SIV-RNA copies/mL) for both TFV-dp (ρ =-0.43, P =0.03, n=21) and FTC-tp (ρ =-0.60, P =0.002, n=21).

During the three months of ART, the LN SIV-RNA levels decayed with a median rate of 0.59 week^-1^ (range: 0.35 to 1.29, n=21) (between M0 and M1: median rate 0.99 week^-1^ (range: 0.14 to 1.5) and between M1 and M3: median rate 0.42 week^-1^ (range: 0.27 to 0.98)). Similarly, rectal SIV-RNA and -DNA levels decayed with ART. Though large sizes of LN SIV-RNA, and rectal SIV-RNA and -DNA at ART initiation were associated with lower LNMC IADM levels one month into ART (**Figure 3**), the tissue viral decay rates were not significantly associated with LNMC IADM levels (at either timepoints or the average) (P =NS, n=21) or with MERs (P =NS, n=12).

Between M1 and M3, viral RNA continued to decrease in LN and rectal tissue (from median 3.82 to 2.18, and 2.17 to 0.9 log_10_ SIV-RNA copies/10^6^ cell equivalent, respectively), however increases in TFV-dp and FTC-tp levels in PBMC or LNMC were not associated with decreases of viral RNA in both LN and rectal tissue (P =NS, n=21).

### The decrease in immune activation from baseline was positively correlated with LNMC active drug-metabolite levels at M1

**Figure 4:**
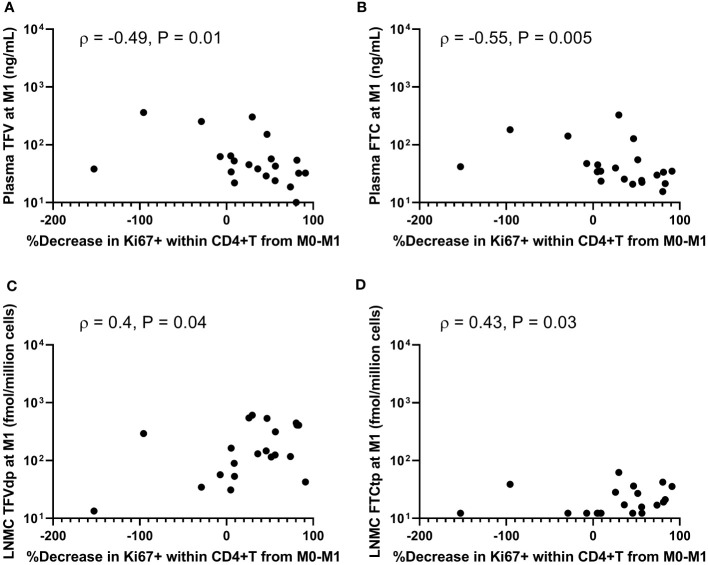
Associations between percentage decreases of Ki67+ levels in peripheral blood CD4+ T-cells during the first month of antiretroviral therapy (ART) and **(A, B)** plasma antiretroviral drug concentrations, and **(C, D)** lymph node mononuclear cells (LNMC) intracellular active drug metabolite levels at month 1 of ART.

No significant association was observed between the fraction of Ki67+ cells within PB CD4+T, CD8+T, and CD20+B cells at M1 and plasma ARV concentrations at M1 (P =NS, n=21). In contrast, %Ki67+ levels at M1 in both PB CD4+T and CD8+T were inversely correlated with PBMC IADM levels at M1 (TFV-dp: ρ ≤-0.43, P <0.03; FTC-tp: ρ ≤-0.47, P <0.02, n=21) and LNMC IADM levels at M1 but only for FTC-tp (ρ ≤ -0.50, P ≤0.01, n=21). Similarly, the %Ki67+ levels at M1 in PB CD4+T, CD8+T, and CD20+B cells were generally negatively correlated with dNTP concentrations in LN tissue at M1 but reached statistical significance only for dCTP (P ≤0.05, n=12). ART initiation reduced immune activation, and the percent decreases of Ki67+ levels in PB CD4+T (and CD8+T and CD20+B; data not shown) during the first month of ART significantly correlated negatively with levels of plasma ARV concentrations at M1 (TFV: ρ =-0.49, P =0.01; FTC: ρ =-0.55, P =0.005, n=21) (**Figures 4A, B**). Though no significant associations were observed between decreases of Ki67+ levels during the first month of ART and PBMC IADM levels at M1, a larger percentage reduction in Ki67+ levels during the first month of ART but only in PB CD4+T trended towards higher LNMC IADM levels at M1 (TFV-dp: ρ =0.4, P =0.04; FTC-tp: ρ =0.43, P =0.03, n=21) (**Figures 4C, D**).

Further changes in %Ki67+ levels between M1 and M3 were not significantly associated with the changes in plasma ARV concentrations or PBMC IADM levels between M1 and M3. Interestingly, the percent decrease of Ki67+ levels in PB CD4+T, CD8+T, and CD20+B between M1 and M3 was positively correlated with the percent increase in LNMC TFV-dp (but not FTC-tp) levels between M1 and M3 (ρ >0.5, P ≤0.01, n=21).

## Discussion

In the 21 SIV-infected RMs of this study, we recently showed no immunotherapeutic effect of anti-α_4_β_7_ mAb infusions on the viral setpoint of SIV-infection (22). The observation in this study that antagonism of α_4_β_7_ integrin had no significant effect on PBMC IADM levels agrees with the finding that blocking the α_4_β_7_ integrin to affect the trafficking of α_4_β_7_+ lymphocytes to gut-associated lymphoid tissue results in a minor perturbation of the PB compartment with a minimal influx of lymphocytes into PB (33). This lack of effect allowed us to increase the sample size and retrospectively study the associations between the dynamics of viral replication and ARV levels in tissues with higher statistical power. Here we observed an increase in the concentration of NRTIs active moieties (IADM) in both lymph nodes and PB between months 1 and 3 of ART, and an inverse association between viral dissemination in plasma/tissues (both LN and rectal tissue) at ART initiation and IADM levels at one month into ART. As NRTIs need to sequentially phosphorylate inside cells by host enzymes to their pharmacologically active triphosphates, we suggest that this inverse association has broad implications for drugs that need to be phosphorylated to exert their pharmacological activity, especially in the settings of pre- or post-exposure prophylaxis targeting highly virulent pathogens replicating in anatomic compartments characterized by high levels of inflammation, such as rectal\vaginal tissues of Truvada-PREP recipients with a high risk of contracting HIV infection (34, 35), or the lungs of Remdesivir\Molnupiravir recipients for the treatment of SARS-CoV-2 infection (36–39).

Earlier studies of daily administration of subcutaneous TFV and FTC in SIV-infected macaques and oral tenofovir disoproxil fumarate (TDF) and FTC in HIV-infected humans agree with the observation in this study of significantly lower TFV-dp and FTC-tp levels in LNMC as compared to PBMC (11–14, 23). One hypothesis to explain the markedly lower LNMC IADM levels is perhaps a poor ARV penetration into LN tissues (40). With a single-dose pharmacokinetic (PK) study in rats and using *in-vivo* positron emission tomography imaging coupled with *ex-vivo* biodistribution, we previously showed that the parent drug concentration of the intravenously injected tenofovir is significantly lower in lymphoid tissues as compared to PB (41). All multiple-dose PK studies on the characterization of ARV distribution in LN tissue, in addition to plasma ARV concentrations, analyzed either isolated mononuclear cells (MNC) or tissue homogenates, but not both. To our knowledge, this is the first multiple-dose study to measure drug levels in LN tissue using both MNC isolation and tissue homogenization methods in nonhuman primates.

The observation that parent NRTI trough concentrations in homogenized LN tissue are several-fold higher than plasma trough concentrations (**Figure 2C** and **Supplementary Table 3**) is consistent with an earlier study that dosed juvenile/adult RMs with daily ARVs for 10 days (16). Hence, the observation of lower LNMC IADM levels as compared to PBMC is perhaps not due to poor cumulative access of the parent drug into the LN tissue. The parent ARV concentration measured in LN homogenate using LC-MS/MS is an aggregate average drug exposure within the analyzed tissue, and studies using mass spectrometry imaging have shown that intratissue spatial ARV drug distribution is heterogenous in distinct cellular and anatomic compartments (7–10). Hence, the presence of the parent drug in tissue does not necessarily mean that it is indeed available for uptake inside LNMC, or enough to reach concentrations required to inhibit viral RNA replication. Yet, in this study, higher ARV concentrations in LN tissue translated to higher active drug moiety concentrations within the tissue (**Supplementary Figure 6**). However, it is worth noting that parent drug measurement in homogenized tissue is the sum of both extracellular and intracellular ARV that exists as the parent drug, and the partition between these two compartments cannot be ascertained with the current analytical methods.

As TFV-dp and FTC-tp are ion-trapped inside the cells, both macaque and human studies have demonstrated that they persist with relatively long intracellular half-lives compared with parent drug in plasma (42–46), and with daily dosing, this pharmacological characteristic leads to a gradual buildup of active drug-metabolites inside the cells to a steady-state concentration (Css). The time to reach Css depends on the intracellular half-life (47); since the half-life of FTC-tp is lower than TFV-dp, from oral PrEP trials in HIV-negative adults (daily TDF/FTC as Truvada), the Css for FTC-tp in PBMC was achieved faster than for TFV-dp [3-7 days for FTC-tp compared to 7-12 days for TFV-dp (42, 45, 48)]. In a PK study that included both HIV-negative and positive participants, both TFV-dp and FTC-tp concentrations in PBMC were generally lower in HIV-positive vs. negative participants at Day 30, although the difference did not achieve statistical significance (42). In a longitudinal study of HIV-1 infected individuals who initiated effective ART at ~500 CD4+ T-cell counts and ~4 Log_10_ PVL, Css was reached by M1, and no significant changes in PBMC and LNMC IADM levels was observed until the end of the study at M6 (12). To our knowledge, only one study of four uninfected macaques showed Css in PBMC reaching in about a week with daily oral TAF/FTC (49). In contrast, in this current RM study, both PBMC and LNMC IADM levels are significantly higher at M3 vs. M1.

Possible reasons for the observed increase in IADM levels between M1 and M3 entail several cellular and pharmacological considerations: (a) cell type/cell subset composition within MNCs, (b) cellular activation state, (c) intracellular kinases responsible for phosphorylation/phosphorylation rate (47), and (d) parent drug availability for uptake into the cells. In order:

(a) Cell-type dependent PK characteristics were previously demonstrated *in-vivo* in which Css for both TFV-dp and FTC-tp in CD14+ cells (monocytes) was reported to be reaching faster and ~3-fold higher compared to Css in T and B lymphocytes (42). One explanation for increasing IADM levels between M1 and M3 is perhaps due to an increasing frequency of monocytes within the MNC population during ART. However, studies in macaques and humans have shown that the proportion of monocytes in PBMC and LNMC during untreated SIV/HIV infection is higher than in controls, and decreases with ART (50, 51). In line with the literature, in the 21 macaques of this study, the percent of monocytes within the combined monocyte and lymphocyte population, which makes up >98% of the MNC population in both PB and lymph nodes (52), was significantly higher at M0 compared to pre-infection levels (mean ± SD: 11.8 ± 4.5% vs. 6.1 ± 4.1%, P <0.001). Though a non-significant decrease was noted by M1 (11.0 ± 6.0%), compared to M0 and M1, the monocyte fraction significantly decreased by M3 (8.8 ± 4.4%, P <0.05). Hence, we excluded the changing proportion of monocyte cell subset during ART as a lone potential factor for the observed increase of IADM levels.
(b) Immune activation reduction during ART changes the fraction of activated vs. resting cells. *In-vitro* studies have shown that the cellular activation state significantly influences the intracellular pharmacology of NRTIs, demonstrating a 2-3 fold higher accumulation of TFV-dp in resting vs. activated cells (53, 54). However, to observe a 1.5-fold increase in IADM levels between M1 and M3, our simulations show that ~65% of the MNCs must be in the activated state at M1, and for a 2-fold increase between M1 and M3, ~90% of the MNCs at M1 must be in the activated state. Hence, we excluded the immune activation reduction during continued ART between M1 and M3 as a sole potential factor for the continued increase of IADM levels.
(c) NRTIs require kinase-mediated phosphorylation inside the cells for pharmacologic activation. Since an increase in the phosphorylation rates should lead to an increase in IADM levels, we employed a simplified version of a TFV-validated PBPK model built by *Perazzolo et al.* (30) as an investigational tool to test the PK hypothesis. Modeling an increase in the phosphorylation rates (*k_f1_* and/or *k_f2_*) coupled with changes in lymphocytes trafficking rates as result of ART-induced reduction in immune activation (55) led to an increase in IADM levels in both LNMC and PBMC, with a steeper growth in the former compartment, as observed in our dataset. This hypothesis would not conflict with the observation that endogenous dNTP concentrations were not statistically significantly different between M1 and M3 because different (or a subset of) kinases with widely differing affinities for their unphosphorylated substrates could be responsible for the formation of active drug-metabolities vs. endogenous nucleotides; indeed, enzymes that could potentially phosphorylate TFV are still under investigation (56). In this scenario, the simulations also predict an accumulation of several-fold higher parent drug levels in the LNs than in the plasma and a plateau reached by month 1 of ART, which appears consistent with the dynamics of parent TFV in LNs found in a subgroup of animals for which this covariate was observed (Subgroup A). This subgroup of animals is however characterized by lower levels of baseline viral loads compared to the full group, a feature that may mask the true changes of parent drug levels during time on ART in LNs.
(d) Using the same model, an alternative mechanism of promoting the accessibility of the ARVs to the cellular compartments within the lymph node could explain the dynamics of plasma TFV and TFV-dp in LNMC and PBMC observed with the full dataset as well as a putative increase in parent drug levels in LNs during time. Simulating an increased availability of parent ARV in the LN for cellular uptake by boosting the LNMC uptake of ARV (*k_in_LNMC_*), and coupled with changes in lymphocytes trafficking rates as result of ART-induced reduction in immune activation, our PBPK model explained the observed increase of LNMC and PBMC IADM levels between M1 and M3. This PK hypothesis, which seems to point to a blockade of the parent drug in the LN from being available for phosphorylation in the early stages of ART, could be tested in future pre-clinical studies by characterizing ARV exposure during prolonged ART using mass spectrometry imaging (57, 58).

Several infection-induced damaging factors and ART-induced rectification of infection-associated damage could contribute to improved drug distribution in the LN or increased drug uptake by LNMC during ART. For example, tissue fibrosis caused by collagen deposition induces structural changes that may affect ARV distribution and perfusion patterns throughout the LN and restrict LNMC’s ability to interact with drugs and its motility (40). Furthermore, TFV was shown to be transported by unspecific endocytosis across lymphocyte membrane (59) and this membrane transport could be affected as a consequence of immune activation levels. TFV has also been shown to be taken up by active transporters, however, the modulation of afflux and efflux transporters of NRTIs following ART-induced reduction in immune activation, or in general, in disease is still largely unknown (60). To our knowledge, the effect of long-term ART on NRTIs active moiety levels in tissues is also unknown. A promising approach to bypass this putative blockade of the parent antivirals in the lymph nodes includes encapsulating the drugs in lipid nanoparticle formulations to improve drug penetration, distribution, and retention in LN tissue viral reservoirs during their first passage from the SC space (19, 20, 61).

The reduced PBPK model of nonhuman primates by *Perazzolo* et al. (30) was critical to testing the hypotheses abovementioned. PBPK modeling has the advantage to use the host physiology (e.g., organ volumes and blood flows) and drug characteristics (e.g., *in-vitro* phosphorylation rates) to test mechanistic hypotheses. Simplifications of the model were carried out to focus on the plasma, PBMC, and LNMC compartments. For this reason, a lymphatic system physiological circulation was included to single out LN-to-blood MNC migrations and lymph node drug extra/intracellular kinetics. Including these factors allowed us to explain our observed data with a certain degree of biological soundness. However, our simplified model does not take into account the various cell types within the MNC population and doesn’t distinguish between the activated vs. resting state of cells. A possible modeling improvement is to dynamically link current MNC levels with ARV pharmacodynamic endpoints, such as viral dissemination, cell counts, and other immunological measurements.

Despite LNMC IADM levels at month one into ART correlated negatively with the level of viral dissemination at ART initiation, higher LNMC IADM levels or higher MERs at M1 did not lead to faster viral decay in plasma or in LN or rectal tissue. One study of chronically HIV-1 infected patients demonstrated that a lower TFV-dp:dATP ratio in PBMC at Day 7 of ART was significantly associated with a reduced viral decay rate (62). The observed MERs at M1 in this study were similar to previous studies (16, 18). Also, the plasma SIV viral load in our study decayed with first and second-phase half-lives (median ~1 day and ~21 days, respectively) comparable to the biphasic decline of plasma HIV-1 RNA in humans initiating ART. Perhaps, the ideal timepoints for MERs measurements to test these associations with viral decay rates are during the first phase of plasma viral decay (first two weeks of ART), but the earliest we measured in this study was on Day 28, and likely too late. Additionally, we did not measure the levels of the integrase strand transfer inhibitor L-870812, and its differential levels could in principle individually modulate the viral decay rates, and/or its presence could mask these associations with other drugs in the regimen. Furthermore, although we present a model of trafficking of lymphocytes between peripheral blood and lymphoid tissues, only peripheral lymph nodes were biopsied. Other lymphoid tissues may differentially contribute to the observed dynamics, nevertheless, daily dosing studies in rhesus macaques have shown that the parent ARV concentrations and their IADM levels in four different LN clusters (axillary, mesenteric, inguinal, and iliac) and the spleen are similar (16, 23). A more robust validation of our observation would however require following the same dynamics in a group of healthy uninfected animals as an ART-only control group. Nevertheless, combining the inverse correlation between plasma viremia at ART initiation and levels of active moieties in LNMC at M1, and the inverse correlation between active moiety levels in LNMC at M1 and the slope of their change from M1 to M3 suggests that an earlier plateau for IADM levels will be reached in healthy uninfected controls.

In conclusion, high levels of viral dissemination at the time of initiation of antiretroviral therapy with nucleos(t)ide reverse transcriptase inhibitors limits the formation of intracellular active drug-metabolites in the LN possibly through one or a combination of two factors: reduced phosphorylation rates of the kinases involved in the formation of active drug moieties and a blockade of the parent drug in the LNs.

## Data availability statement

The original contributions presented in the study are included in the article/**Supplementary material**. Further inquiries can be directed to the corresponding author.

## Ethics statement

The animal study was approved by National Institute of Allergy and Infectious Diseases (NIAID) Animal Care and Use Committee. The study was conducted in accordance with the local legislation and institutional requirements.

## Author contributions

SS, SP, RH, and MDM designed the research. SS, PD, SP, AB, AT, JK, HJ, RT, MD, MC, and MDM performed the research. SS wrote the manuscript. SS, SP, HJ, and MDM made the figures and tables. All authors analyzed and interpreted the data and critically reviewed the manuscript. MDM provided oversight and helped with the editing of the manuscript. All authors contributed to the article and approved the submitted version.
