# Supplementary material for: Viral dissemination and immune activation modulate antiretroviral drug levels in lymph nodes of SIV-infected rhesus macaques
Source: Front Immunol. 2023 Sep 18;14:1213455. doi: 10.3389/fimmu.2023.1213455 (PMC10544331; doi:10.3389/fimmu.2023.1213455)
Supplement: Supplementary file 1 [file DataSheet_1.docx]

**Supplementary material**

**Viral dissemination and immune activation modulate antiretroviral drug levels in lymph nodes of SIV-infected rhesus macaques**

**Sharat Srinivasula^1*^, Paula Degrange^2^, Simone Perazzolo^3^, Andrew Bonvillain^2^, Amanda Tobery^2^, Jacob Kaplan^4^, Hyukjin Jang^1^, Refika Turnier^5^, Michael Davies^5^, Mackenzie Cottrell^6^, Rodney J.Y. Ho^3,7^, Michele Di Mascio^4^**

^1^AIDS Imaging Research Section, Clinical Monitoring Research Program Directorate, Frederick National Laboratory for Cancer Research, Frederick, Maryland, USA

^2^AIDS Imaging Research Section, Charles River Laboratories, Integrated Research Facility, NIAID, NIH, Frederick, Maryland, USA

^3^Department of Pharmaceutics, University of Washington, Seattle, WA, USA

^4^AIDS Imaging Research Section, Division of Clinical Research, NIAID, NIH, Poolesville, Maryland, USA

^5^Clinical Support Laboratory, Frederick National Laboratory for Cancer Research, Frederick, Maryland, USA

^6^University of North Carolina Eshelman School of Pharmacy, Chapel Hill, North Carolina, USA

^7^Department of Bioengineering, University of Washington, Seattle, WA, USA

**Corresponding author:** Sharat Srinivasula

5601 Fishers Lane, Room 4D29, Rockville, MD 20852
Phone: 240-669-5261

[**srinivasulas@mail.nih.gov**](mailto:srinivasulas@mail.nih.gov)

**Model Glossary:**

V: volume (mL)

C: concentration (ng/mL)

D: drug dose (ng)

Q: flow rate (mL/min)

CL: clearance (mL/min)

k: kinetic rate constant (1/min)

subQ: subcutaneous

inj: injection

p: plasma

rob: rest of body

PBMC: peripheral blood mononuclear cells

LNMC: lymph node mononuclear cells

in/out: drug uptake/release into/out of the cell

K_abs_: blood absorption rate

K_kidney or rob_: kidney or body partition

sin: lymph node sinus

f: forward (phosphorylation)

b: backward (dephosphorylation)

migration in/out: cell migration in/out of lymph nodes from/to blood

mp: monophosphate

dp: diphosphate

**Model Equations**

**Subcutaneous compartment**

$\dot{C_{subQ}}= -C_{subQ}k_{abs}$ | $C_{subQ}(0)=\frac{D}{V_{inj}}$ (1)

The initial condition for the subcutaneous compartment was equated to the bolus drug dose divided by the injection volume. From the second day onwards, the initial condition for the subcutaneous compartment was set to the bolus drug dose divided by the injection volume (the new dose for the day) plus the end-of-interval concentration from the day before. The initial conditions for the rest of the ordinary differential equations (eq. 2-11) are set to zero.

**Plasma compartment**

$V_{p}\dot{C_{p}}=C_{subQ}k_{abs}V_{inj}-Q_{kidney}C_{p}+\frac{Q_{kidney}C_{kidney}}{K_{kidney}}-Q_{rob}C_{p}+\frac{{(Q}_{rob}-Q_{lymph})C_{rob}}{K_{rob}}+Q_{lymph}C_{sin}- k_{in,PBMC}V_{p}C_{p}+ k_{out,PBMC}V_{PBMC}C_{PBMC}$ (2)

**Kidney**

$V_{kidney}\dot{C_{kidney}}=Q_{kidney}C_{p}-\frac{Q_{kidney}C_{kidney}}{K_{kidney}}-\frac{C_{kidney}CL}{K_{kidney}}$ (3)

**Rest-of-body**

$V_{rob}\dot{C_{rob}}=Q_{rob}C_{p}-\frac{{(Q}_{rob}-Q_{lymph})C_{rob}}{K_{rob}}-\frac{Q_{lymph}C_{rob}}{K_{rob}}$ (4)

The ‘kidney’ and ‘Rest-of-body’ are modeled as flow-limited compartments. For more details about the model, see Perazzolo et al (1). Clearance (CL) was applied to the equilibrating venous blood on the kidney as a PBPK perfusion-limited organ. K_rob_ was assumed to be the same also in the interstitial fluid driving the lymphatic uptake. This was demonstrated in Perazzolo et al (1) for the full tenofovir model. Tenofovir should be modeled as a membrane-limited distribution in Rest-of-body (assuming muscles are dominant) to characterize the biphasic decay in the plasma and tissues. But for simplicity, here we assumed that Rest-of-body behaves as a perfusion-limited organ (i.e., rapid equilibration). In this approximation, we evaluated that the error is small for the scope of this study. We also collapsed the whole-body lymphatic system of Perazzolo et al., into a single representative compartment as the scope of this simplified model is to understand the general mechanisms that can explain the observations.

**PBMC**

TFV (parent)

$V_{PBMC}\dot{C_{PBMC}}= k_{in,PBMC}V_{p}C_{p}- k_{out,PBMC}V_{PBMC}C_{PBMC}- k_{f1}V_{PBMC}C_{PBMC}+ k_{b1}V_{PBMC}C_{PBMC,mp}+k_{migration,out}V_{LNMC}C_{LNMC} {- k}_{migration,in}V_{PBMC}C_{PBMC}$ (5)

TFV-mp

$V_{PBMC}\dot{C_{PBMC,mp}}= k_{f1}V_{PBMC}C_{PBMC}-k_{b1}V_{PBMC}C_{PBMC,mp}-k_{f2}V_{PBMC}C_{PBMC,mp}+k_{b2}V_{PBMC}C_{PBMC,dp}+k_{migration,out}{V_{LNMC}C}_{LNMC,mp}-k_{migration,in}V_{PBMC}C_{PBMC,mp}$ (6)

TFV-dp

$V_{PBMC}\dot{C_{PBMC,dp}}=k_{f2}V_{PBMC}C_{PBMC,mp}-k_{b2}V_{PBMC}C_{PBMC,dp}+k_{migration,out}V_{LNMC}C_{LNMC,dp}-k_{migration,in}V_{PBMC}C_{PBMC,dp}$ (7)

**Sinus compartment**

$V_{sin}\dot{C_{sin}}=\frac{Q_{lymph}C_{rob}}{K_{rob}}-Q_{lymph}C_{sin}-k_{in,LNMC}V_{sin}C_{sin}+k_{out,LNMC}V_{LNMC}C_{LNMC}$ (8)

**LNMC**

TFV (parent)

$V_{LNMC}\dot{C_{LNMC}}=k_{in,LNMC}V_{sin}C_{sin}-k_{out,LNMC}V_{LNMC}C_{LNMC}-k_{f1}V_{LNMC}C_{LNMC}+k_{b1}V_{LNMC}C_{LNMC,mp}-k_{migration,out}V_{LNMC}C_{LNMC}+k_{migration,in}V_{PBMC}C_{PBMC}$ (9)

TFV-mp

$V_{LNMC}\dot{C_{LNMC,mp}}={k_{f1}V}_{LNMC}C_{LNMC}-k_{b1}V_{LNMC}C_{LNMC, mp}-k_{f2}V_{LNMC}C_{LNMC,mp}+k_{b2}V_{LNMC}C_{LNMC,dp}-k_{migration,out}V_{LNMC}C_{LNMC,mp}+k_{migration,in}V_{PBMC}C_{PBMC,mp}$ (10)

TFV-dp

$V_{LNMC}\dot{C_{LNMC, dp}}={k_{f2}V}_{LNMC}C_{LNMC,mp}-k_{b2}V_{LNMC}C_{LNMC,dp}-k_{migration,out}{V_{LNMC}C}_{LNMC,dp}+k_{migration,in}V_{PBMC}C_{PBMC,dp}$ (11)

An average cell volume of 282.9 femtoL (2), and molecular weight of 447.17g/mol for TFV-dp was used for the conversion of TFV-dp concentration between ng/mL and fmol/million cells.

To resemble a physiological change in *CL*, k_in_LNMC_, *k_f1_*, *k_migration,in_*, and *k_migration,out_* parameters with time, an empirical sigmoid time-varying function was used and is given by:

$$Y=Bottom+ \frac{(Top-Bottom)}{1+e^{-S*(t-HW)}}$$

Where,

*Bottom* is the Y-value at ART initiation,

*Top* is the Y-value at month 3 of ART,

*S* is the steepness (slope) of the curve, and

*HW* is the time (t) when the parameter change is halfway between the *Bottom* and *Top*.

The halfway value of 45 days and a slope of 0.12 was used.

**Supplementary results:**

***LN tissue contained more parent drug than intracellular active drug-metabolite (Subgroup A analysis)***

Of the 25 homogenized LN tissue samples (n=12 at M1 and n=13 at M3) analyzed for drug active moiety concentrations (expressed as pmol/g of tissue), two animals at M1 for FTC-tp measured below LLOQ, and these samples were imputed at LLOQ. Expressed in molar concentration to account for their molecular weights, between the two forms (parent ARV and their active moieties), a vast proportion of the drug in LN tissue at M1 was present as parent drug, with a minimal proportion converted to the active drug-metabolite (TFV-dp: median 13.5% (range: 4.9 to 24.7); FTC-tp: median 6.9% (range: 1.9 to 11.3)). Compared to M1, at M3 there was a trend towards an increased proportion of TFV-dp (median 24.2% (range: 1.4 to 42.9), P =0.08), but a decreased proportion of FTC-tp (median 2.3% (range: 0.3 to 9.0), P =0.01) concomitant to an increase of parent FTC in tissue. Regardless of the timepoint, a significant direct association was observed between the parent drug and their active moiety concentrations within the LN tissue homogenate (Suppl Fig 6). Between the two ARVs, the fraction of the drug that converted to active drug-metabolite was significantly lower for FTC compared to TFV at both M1 (P =0.015) and M3 (P =0.001).

**Suppl. Fig 1**

**

**

**Suppl. Fig. 1.** The study design. The timepoints of lymph node and rectal biopsies (red arrows) for evaluations of antiretroviral drug levels and/or SIV-RNA and SIV-DNA in tissues, and the timepoints of peripheral blood collection (purple arrows) for evaluations of antiretroviral drug levels are indicated along with intravenous infusions of 50mg/kg of either a primatized anti-α_4_β_7_ monoclonal antibody (mAb) (n=11) or an isotype-matched control IgG mAb (n=10) (blue arrows).

**Suppl. Fig 2**

**
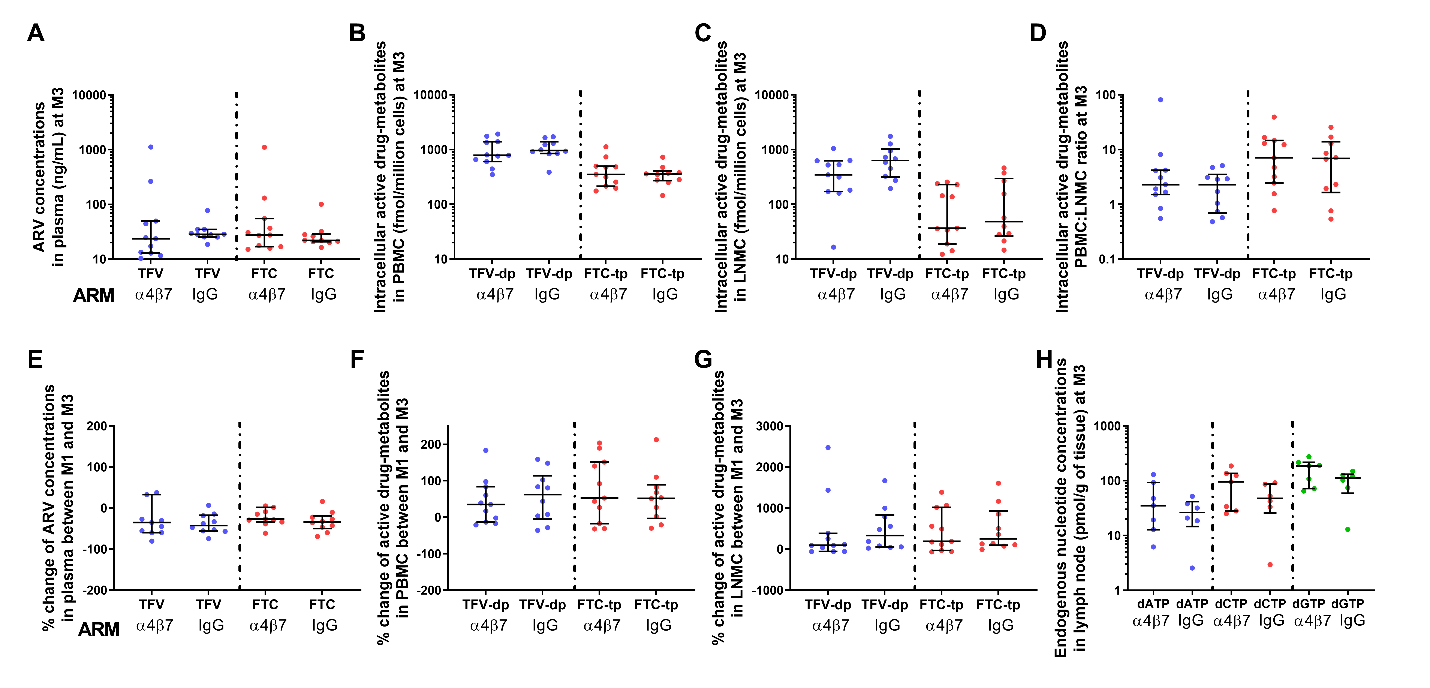
**

**Suppl. Fig. 2.** Comparison between α_4_β_7_  (n=11) and control IgG (n=10) arms of (**A**) Plasma ARV concentrations, and intracellular active drug metabolite (IADM) levels in (**B)** peripheral blood mononuclear cells (PBMC) and (**C**) lymph node mononuclear cells (LNMC), and (**D**) their ratios at month 3 of antiretroviral therapy (ART), the percentage changes in (**E**) plasma ARV concentrations, and IADM levels in (**F**) PBMC and (**G**) LNMC between months 1 and 3 of ART, and (**H**) endogenous nucleotide concentrations (n=7 (α_4_β_7_) vs. 6 (IgG)) at month 3 of ART. No statistically significant effect of the three anti-α_4_β_7_ mAb infusions administered between months 1 and 3 was observed on the drug levels or endogenous nucleotide concentrations. Data were summarized with a scatter dot plot with median and interquartile range.

**Suppl. Fig 3**

**
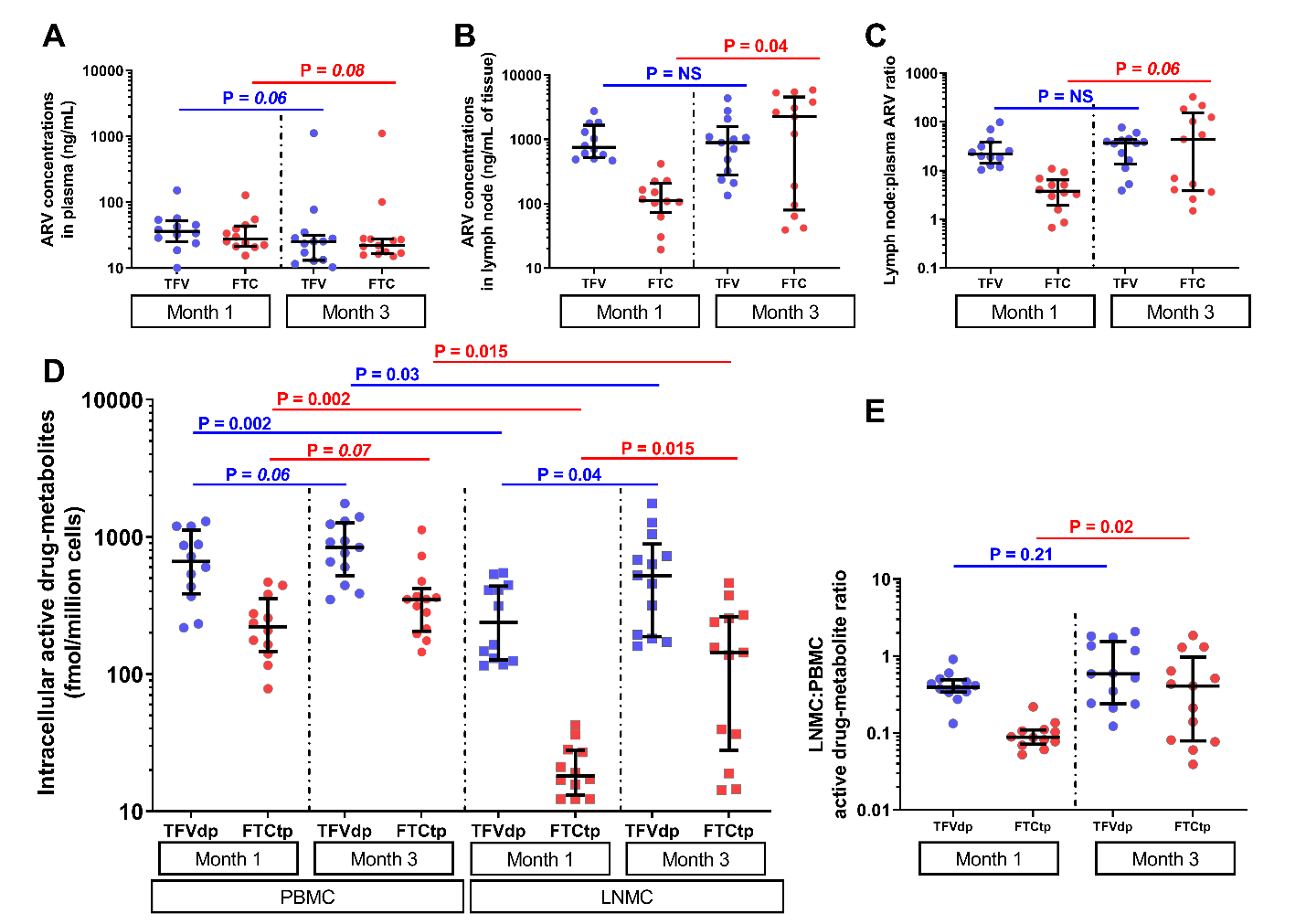
**

**Suppl. Fig. 3.** Parent antiretroviral (ARV) concentrations in (**A**) plasma and (**B**) lymph node tissue homogenate, (**C**) lymph node tissue to plasma ARV ratios, (**D**) the levels of intracellular active drug metabolites (IADM) in peripheral blood mononuclear cells (PBMC) and lymph node mononuclear cells (LNMC), and (**E**) LNMC to PBMC IADM ratios in subgroup A animals (n=12 at M1 and n=13 at M3) at months 1 and 3 of ART. The lymph node tissue ARV concentrations were several-fold higher than plasma ARV concentrations (P <0.005, n=12) and the IADM levels in LNMC were statistically significantly lower than in PBMC (P ≤0.03, n=12). Data were summarized with a scatter dot plot with median and interquartile range.

**Suppl. Fig 4**

**
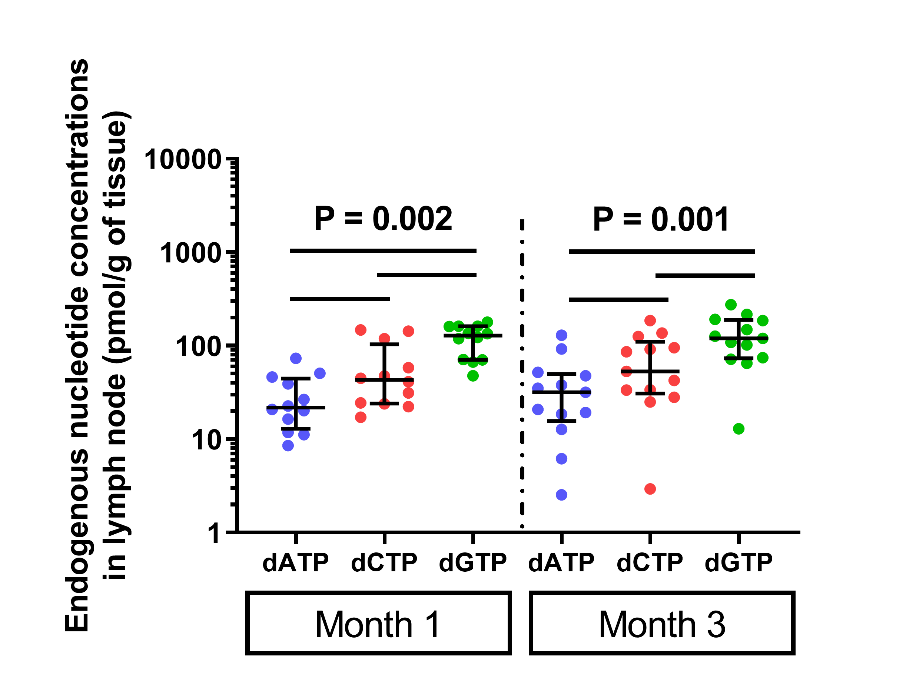
**

**Suppl. Fig. 4.** Concentrations of deoxyadenosine triphosphate (dATP), deoxycytidine triphosphate (dCTP), and deoxyguanosine triphosphate (dGTP) from lymph node tissue homogenates at month 1 (N = 12) and month 3 (N = 13) of antiretroviral therapy (ART) (Subgroup A). No statistically significant changes in all three endogenous nucleotide concentrations were observed between months 1 and 3 of ART (P > 0.53).

**Suppl. Fig 5**

**

**

**Suppl. Fig. 5.** Associations between active drug-metabolite concentrations (TFV-dp and FTC-tp) and endogenous nucleotide concentrations (dATP, dCTP, and dGTP) in lymph node tissue homogenate (Subgroup A). At both months 1 and 3 of antiretroviral therapy, the drug active moiety concentrations generally correlated positively with endogenous nucleotide concentrations.

**Suppl. Fig 6**

**
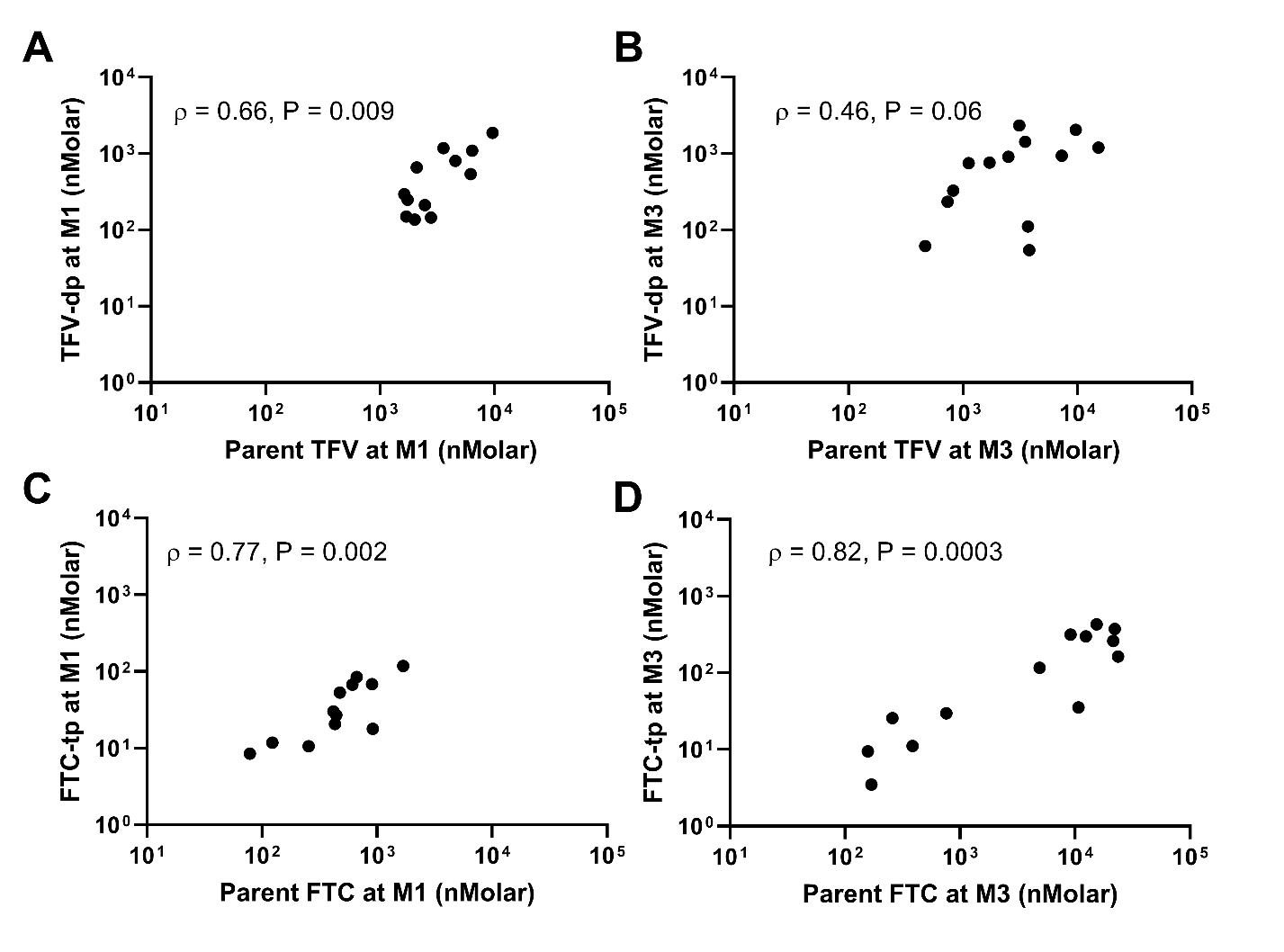
**

**Suppl. Fig. 6.** Associations between parent antiretroviral drug and their active drug-metabolite concentrations within the lymph node tissue at (**A, C**) months 1 (n =12) and (**B, D**) 3 (n =13) of antiretroviral therapy in Subgroup A animals.

**Suppl. Table 1.** Sample sizes for each analyte and for each matrix.

| **# of Animals** | **Parent ARV** | | **Intracellular active drug-metabolites** | | **Endogenous nucleotides** | |
| --- | --- | --- | --- | --- | --- | --- |
| **Analyte** | TFV, FTC | | TFV-dp, FTC-tp | | dATP, dCTP, dGTP | |
| **Time point** | Month 1 | Month 3 | Month 1 | Month 3 | Month 1 | Month 3 |
| **PLASMA** | 21 | 21 |  |  |  |  |
| **PBMC** |  |  | 21 | 21 |  |  |
| **LNMC** |  |  | 21 | 21 |  |  |
| **Lymph node tissue homogenate** | 12 | 13 | 12 | 13 | 12 | 13 |

**Suppl. Table 2.** Median (inter-quartile range) active drug-metabolite levels in peripheral blood mononuclear cells (PBMC), lymph node mononuclear cells (LNMC), and their respective ratios in the full dataset of n=21 animals.

|  |  | **Month 1** | | **Month 3** | |
| --- | --- | --- | --- | --- | --- |
|  | Units | TFV-dp | FTC-tp | TFV-dp | FTC-tp |
| **PBMC** | fmol/million cells | 681.2 (484.9, 1097.3) | 235.3 (158.3, 378.9) | 916.1 (709.7, 1370.8) | 351.1 (252.3, 479.0) |
| **LNMC** | fmol/million cells | 130.9 (54.8, 409.1) | 16.9 (12.3, 31.9) | 522.1 (233.2, 701.3) | 39.5 (24.8, 234.1) |
| **LNMC:PBMC drug-metabolite ratio** | unitless | 0.34 (0.09, 0.45) | 0.08 (0.06, 0.11) | 0.44 (0.24, 1.06) | 0.14 (0.07, 0.47) |

**Suppl. Table 3.** Median (inter-quartile range) parent antiretroviral (ARV) concentrations in blood plasma, lymph node tissue homogenate, and their respective ratios.

|  |  |  | **Month 1** | | **Month 3** | |
| --- | --- | --- | --- | --- | --- | --- |
|  | **Units** | **Sample size** | **TFV** | **FTC** | **TFV** | **FTC** |
| **Plasma** | ng/mL | Full dataset  (n = 21) | 42.9 (30.6, 63.6) | 34.9 (23.9, 51.3) | 27.9 (18.0, 39.8) | 26.2 (18.9, 34.4) |
|  |  | Subgroup A  (n = 12 at M1 and n = 13 at M3) | 36.2 (25.2, 52.0) | 27.8 (21.6, 43.8) | 25.4 (13.2, 31.8) | 22.1 (16.7, 27.9) |
| **Lymph node tissue homogenate** | ng/mL | Subgroup A  (n = 12 at M1 and n = 13 at M3) | 754.9 (520.1, 1664.8) | 113.3 (73, 208.6) | 892.1 (279.4, 1596.2) | 2257.9 (79.9, 4572) |
| **Lymph node:Plasma ARV ratio** | unitless | Subgroup A  (n = 12 at M1 and n = 13 at M3) | 21.8 (14.1, 38.3) | 3.8 (1.9, 6.4) | 36.5 (13.8, 43.2) | 43.9 (3.9, 153.3) |

**Suppl. Table 4.** Median (inter-quartile range) intracellular active drug-metabolite and endogenous nucleotide concentrations in lymph node tissue homogenate, and their respective ratios (MERs) in subgroup A animals (n = 12 at Month 1 and n = 13 at Month 3).

|  | **Units** | **Analyte** | **Month 1** | **Month 3** |
| --- | --- | --- | --- | --- |
| **Active drug-metabolites** | pmol/g | **TFV-dp** | 403.9 (160.2, 987.6) | 739.9 (167.5, 1273.6) |
|  |  | **FTC-tp** | 27.8 (12.9, 66.0) | 112.8 (17.8, 297.7) |
| **Endogenous nucleotides** | pmol/g | **dATP** | 21.8 (13.0, 44.5) | 31.7 (15.7, 49.8) |
|  |  | **dCTP** | 43.0 (24.0, 103.4) | 52.9 (30.8, 110.1) |
|  |  | **dGTP** | 128.3 (70.5, 161.0) | 119.9 (73.3, 188.5) |
| **Active drug-metabolite:Endogenous  nucleotide ratio (MERs)** | unitless | **TFV-dp:dATP** | 15.9 (9, 24.5) | 24.5 (16, 45.8) |
|  |  | **FTC-tp:dCTP** | 0.5 (0.4, 1.1) | 1.1 (0.6, 3.5) |

**Suppl. Table 5.** The physiological and pharmacokinetic parameters and their values used for the PBPK model simulation of TFV in a typical 7.8 kg rhesus macaque. The parameter values were taken from Perazzolo et al (1).

| **Independent parameters** | **At ART initiation** | **At month 3 of ART** | |  |
| --- | --- | --- | --- | --- |
|  |  | Scenario i. Increased kinase-mediated phosphorylation rates | Scenario ii. Increased LNMC uptake of TFV | **Units** |
| Body weight | 7.8 | | | kg |
| ARV dose | 20 | | | mg/kg |
| V_inj_ | 10 | | | mL |
| V_plasma_ | 544 | | | mL |
| V_lymphnodes_ | 58 | | | mL |
| V_kidney_ | 42 | | | mL |
| Clearance (CL) | 112 | 128 | | mL/min |
| Q_kidney_ | 395 | | | mL/min |
| k_in_PBMC_ | 3.00E-05 | | | min^-1^ |
| k_out_PBMC_ | 4.00E-04 | | | min^-1^ |
| k_in_LNMC_ | 2.00E-05 | 2.00E-05 | 1.20E-03 | min^-1^ |
| k_out_LNMC_ | 4.00E-04 | | | min^-1^ |
| k_f1_ | 0.0045 | 0.01 | 0.0045 | min^-1^ |
| k_b1_ | 0.05 | | | min^-1^ |
| k_f2_ | 0.25 | | | min^-1^ |
| k_b2_ | 0.066 | | | min^-1^ |
| k_migration_in_ | 4.00E-03 | 7.50E-03 | | min^-1^ |
| k_migration_out_ | 1.75E-03 | 2.50E-03 | | min^-1^ |
| k_abs_ | 0.03 | | | min^-1^ |
| K_kidney_ | 10 | | | unitless |
| K_rob_ | 3.32 | | | unitless |
| **Dependent parameters** |  | | |  |
| V_sin_ | 0.3*V_lymphnodes_ | | | mL |
| V_LNMC_ | 0.7*V_lymphnodes_ | | | mL |
| V_PBMC_ | 0.01*V_plasma_ | | | mL |
| V_rob_ | (Body weight*1000/1.03)-V_kidney_-V_plasma_ | | | mL |
| Q_lymph_ | 3*(Body weight/10)^0.75^ | | | mL/min |
| Q_rob_ | 2081.4-Q_kidney_ | | | mL/min |

**References**

1. Perazzolo S, Shireman LM, Shen DD, Ho RJY. Physiologically Based Pharmacokinetic Modeling of 3 HIV Drugs in Combination and the Role of Lymphatic System after Subcutaneous Dosing. Part 1: Model for the Free-Drug Mixture. J Pharm Sci. 2022;111(2):529-41.

2. Simiele M, D'Avolio A, Baietto L, Siccardi M, Sciandra M, Agati S, et al. Evaluation of the mean corpuscular volume of peripheral blood mononuclear cells of HIV patients by a coulter counter to determine intracellular drug concentrations. Antimicrob Agents Chemother. 2011;55(6):2976-8.
